# Exciton diamagnetic shifts and valley Zeeman effects in monolayer WS_2_ and MoS_2_ to 65 Tesla

**DOI:** 10.1038/ncomms10643

**Published:** 2016-02-09

**Authors:** Andreas V. Stier, Kathleen M. McCreary, Berend T. Jonker, Junichiro Kono, Scott A. Crooker

**Affiliations:** 1 National High Magnetic Field Laboratory, Los Alamos National Laboratory, Los Alamos, New Mexico 87545, USA; 2 Materials Science and Technology Division, Naval Research Laboratory, Washington, Washington DC 20375, USA; 3 Department of Electrical and Computer Engineering, Rice University, Houston, Texas 77005, USA; 4 Department of Physics and Astronomy, Rice University, Houston, Texas 77005, USA; 5 Department of Materials Science and NanoEngineering, Rice University, Houston, Texas 77005, USA

## Abstract

In bulk and quantum-confined semiconductors, magneto-optical studies have historically played an essential role in determining the fundamental parameters of excitons (size, binding energy, spin, dimensionality and so on). Here we report low-temperature polarized reflection spectroscopy of atomically thin WS_2_ and MoS_2_ in high magnetic fields to 65 T. Both the A and B excitons exhibit similar Zeeman splittings of approximately −230 μeV T^−1^ (*g*-factor ≃−4), thereby quantifying the valley Zeeman effect in monolayer transition-metal disulphides. Crucially, these large fields also allow observation of the small quadratic diamagnetic shifts of both A and B excitons in monolayer WS_2_, from which radii of ∼1.53 and ∼1.16 nm are calculated. Further, when analysed within a model of non-local dielectric screening, these diamagnetic shifts also constrain estimates of the A and B exciton binding energies (410 and 470 meV, respectively, using a reduced A exciton mass of 0.16 times the free electron mass). These results highlight the utility of high magnetic fields for understanding new two-dimensional materials.

## Abstract (web summary)

The spin and valley degrees of freedom are coupled in two-dimensional transition-metal dichalcogenides. Here, the authors use high magnetic fields to optically measure the valley Zeeman effect and diamagnetic exciton shift in tungsten disulphide and molybdenum disulphide, and determine the exciton binding energies.

## Graphical abstract

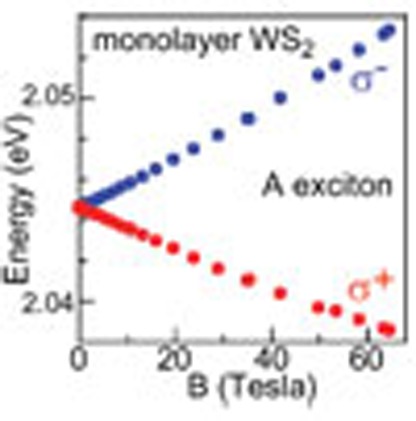

Atomically thin crystals of the transition-metal disulphides (MoS_2_ and WS_2_) and diselenides (MoSe_2_ and WSe_2_) constitute a novel class of monolayer semiconductors that possess direct optical bandgaps located at the degenerate *K* and *K*′ valleys of their hexagonal Brillouin zones^1^,^2^. The considerable recent interest in these two-dimensional (2D) transition-metal dichalcogenides (TMDs) derives from their strong spin–orbit coupling and lack of structural inversion symmetry, which, together with time-reversal symmetry, couples spin and valley degrees of freedom and leads to valley-specific optical selection rules^3^,^4^,^5^,^6^,^7^,^8^: light of *σ*^+^ circular polarization couples to inter-band exciton transitions in the *K* valley, while the opposite (*σ*^−^) circular polarization couples to transitions in the *K*′ valley. The ability to populate and/or probe electrons and holes in specific valleys using polarized light has renewed long-standing interests^8^,^9^,^10^,^11^ in understanding and exploiting such ‘valley pseudospin' degrees of freedom for both fundamental physics and far-reaching applications in, for example, quantum information processing.

The bands and optical transitions at the *K* and *K*′ valleys are nominally degenerate in energy and related by time-reversal symmetry. However, in analogy with conventional spin degrees of freedom, this *K*/*K*′ valley degeneracy can be lifted by external magnetic fields *B*, which break time-reversal symmetry. Recent photoluminescence studies of the monolayer diselenides MoSe_2_ and WSe_2_ in modest fields have indeed demonstrated this ‘valley Zeeman effect', and revealed an energy splitting between *σ*^+^ and *σ*^−^ polarized photoluminescence from the lowest-energy ‘A' exciton transition^12^,^13^,^14^,^15^,^16^,^17^. In most cases, valley splittings in these monolayer diselenides were found to increase linearly with field at a rate of approximately −4*μ*_B_ (≡−232 μeV T^−1^), where *μ*_B_=57.9 μeV T^−1^ is the Bohr magneton. While this value agrees surprisingly well with simple expectations from a two-band tight-binding model (namely, that electron and hole masses are equal, and that the exciton Zeeman shifts derive solely from the hybridized 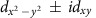 atomic orbitals with magnetic moment ±2*μB* that comprise the *K*/*K*′ valence bands^12^,^13^,^14^,^15^), it is also widely appreciated that a more complete treatment based on established **k**·**p** theory should, with proper inclusion of strong excitonic effects, also provide an accurate description. However, initial **k**·**p** models have so far had limited success accounting for the measured valley Zeeman effect in monolayer TMDs^12^,^16^,^18^,^19^.

Regardless of circumstances, magneto-optical studies of these new monolayer semiconductors are still at a relatively early stage, and several outstanding questions remain. In particular, measurements of valley Zeeman effects in the monolayer disulphides WS_2_ and MoS_2_ have not been reported to date, which would provide a natural complement to the existing data on monolayer WSe_2_ and MoSe_2_. In addition, the valley Zeeman splitting of the higher-energy ‘B' exciton has not yet been reported in any of these 2D materials. Both of these studies would provide a more complete experimental basis against which to benchmark new theoretical approaches. And finally, the diamagnetic energy shift of these excitons, which is anticipated to increase quadratically with field and from which the spatial extent of the fundamental (1*s*) exciton wavefunctions can be directly inferred^20^,^21^,^22^, has not yet been observed in any of the monolayer TMDs. Likely, this is because the diamagnetic shift, Δ*E*_dia_=*e*^2^〈*r*^2^〉_1*s*_*B*^2^/8*m*_r_, is expected to be very small and difficult to spectrally resolve in these materials owing to the small root mean squared (r.m.s.) radius of the 1*s* exciton 
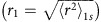
, and large reduced mass 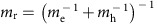. For example, if *r*_1_≈1.5 nm and *m*_e_=*m*_h_≈*m*_0_/2 (where *m*_0_ is the bare electron mass and *m*_e/h_ is the effective electron/hole mass), then Δ*E*_dia_ is only ∼20 μeV at *B*=10 T, clearly motivating the need for large magnetic fields. Crucially, knowledge of Δ*E*_dia_ can also constrain estimates of the exciton binding energy—a subject of considerable recent interest in the monolayer TMDs^23^,^24^,^25^,^26^,^27^,^28^,^29^,^30^,^31^,^32^,^33^,^34^,^35^, wherein the effects of non-local dielectric screening and Berry curvature can generate a markedly non-hydrogenic Rydberg series of exciton states and associated binding energies^36^,^37^,^38^,^39^,^40^.

In the following, we address these questions with a systematic study of circularly polarized magneto-reflection from large-area films of monolayer WS_2_ and MoS_2_ at low temperatures (4 K) and in very high pulsed magnetic fields up to 65 T. Clear valley splittings of about −230 μeV T^−1^ are observed for both the A and B excitons, providing measurements of the valley Zeeman effect and associated *g*-factors in monolayer transition-metal disulphides. Moreover, due to the very large magnetic fields used in these studies, we are also able to resolve the small quadratic diamagnetic shifts of both A and B excitons in monolayer WS_2_ (0.32±0.02 and 0.11±0.02 μeV T^−2^, respectively), permitting estimates of the r.m.s. exciton radius *r*_1_. These results are compared with similar measurements of bulk WS_2_ crystals, and are quantitatively modelled within the context of the non-hydrogenic binding potential^23^,^36^,^37^ that is believed to exist in 2D semiconductors due to non-local dielectric screening. Within this framework, we estimate A and B exciton binding energies of ∼410 and ∼470 meV, respectively, and we show how these values scale with reduced mass *m*_r_.

## Results

### Samples and experimental set-up

Large-area samples of monolayer WS_2_ and MoS_2_ were grown by chemical vapour deposition on SiO_2_/Si substrates^41^,^42^. MoO_3_ and pure sulphur powder were used as precursor and reactant materials, respectively, and the growth was performed at a reactant temperature of 625 °C. In addition, perylene-3,4,9,10-tetracarboxylic acid tetrapotassium salt was loaded on the SiO_2_/Si substrate, which acted as a seeding promoter to achieve uniform large-area monolayer crystals^43^. The monolayer nature and high quality of these samples were confirmed by photoluminescence and Raman studies^42^ (Supplementary Figs 1–5 and Supplementary Notes 1–5). In addition, a freshly exfoliated surface of a bulk WS_2_ crystal was also prepared.

Magneto-reflectance studies were performed at cryogenic temperatures (down to 4 K) in a capacitor-driven 65 T pulsed magnet at the National High Magnetic Field Laboratory in Los Alamos. Broadband white light from a xenon lamp was coupled to the samples using a 100 μm diameter multimode optical fibre. The light was focused onto the sample at near-normal incidence using a single aspheric lens, and the reflected light was refocused by the lens into a 600 μm diameter collection fibre. A thin-film circular polarizer mounted over the delivery or collection fibre provided *σ*^+^ or *σ*^−^ circular polarization sensitivity. The collected light was dispersed in a 300 mm spectrometer and detected with a charge-coupled device detector. Spectra were acquired continuously at a rate of 500 Hz throughout the ∼50 ms long magnet pulse.

### Exciton transitions and Zeeman effects in monolayer TMDs

**Figure 1:**
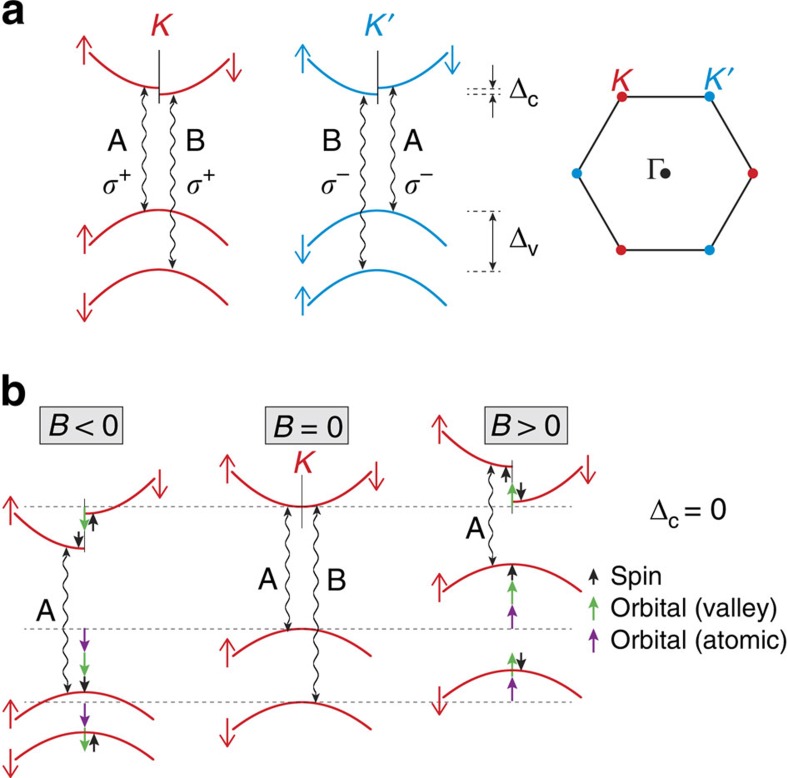
Excitonic transitions and Zeeman shifts in monolayer TMDs. (**a**) Diagrams of the conduction and valence bands at the *K* and *K*′ valleys of the monolayer transition-metal dichalcogenides, showing the A and B exciton transitions (wavy lines) and the associated valley-specific optical selection rules for *σ*^+^ and *σ*^−^ light. For clarity, the spin-up and spin-down conduction bands are separately drawn on the left and right sides within each valley, respectively. A small spin–orbit splitting in the conduction band, Δ_c_, is also depicted for completeness (theory predicts Δ_c_∼+30 and −5 meV for WS_2_ and MoS_2_, respectively^45^). (**b**) Diagrams depict the relative shifts of the conduction and valence bands in the *K* valley (that is, *σ*^+^ transitions) due to applied magnetic fields 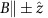. For clarity, Δ_c_=0 here. The contributions to each band's Zeeman shift from spin, atomic orbital and valley orbital (Berry curvature) magnetic moment are depicted separately by black, purple and green arrows. The *σ*^+^ polarized A and B exciton transition energies decrease (increase) in positive (negative) field. By time-reversal symmetry, the shifts depicted here for *B*<0 in the *K* valley are equivalent to those in the *K*′ valley (*σ*^−^ transitions) when *B*>0.

Figure 1a depicts a single-particle energy diagram of the conduction and valence bands in monolayer TMDs at the *K* and *K*′ points of the hexagonal Brillouin zone, along with the A and B exciton transitions (wavy lines) and valley-specific optical selection rules. Strong spin–orbit coupling of the valence band splits the spin-up and spin-down components (by Δ_v_ ∼400 and 150 meV in WS_2_ and MoS_2_, respectively), giving rise to the well-separated A and B exciton transitions that are observed in optical absorption or reflection spectra. As depicted, *σ*^+^ circularly polarized light couples to both the A and B exciton transitions in the *K* valley, while light of the opposite *σ*^−^ circular polarization couples to the exciton transitions in the *K*′ valley.

At zero magnetic field, time-reversed pairs of states in the *K* and *K*′ valleys—for example, spin-up conduction (valence) bands in *K* and spin-down conduction (valence) bands in *K*′—necessarily have the same energy and have equal-but-opposite total magnetic moment 
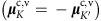
. Therefore, an applied magnetic field, which breaks time-reversal symmetry, will lift the *K*/*K*′ valley degeneracy by shifting time-reversed pairs of states in opposite directions in accord with the Zeeman energy −***μ***·**B**. This will Zeeman shift the measured exciton energy if the relevant conduction and valence band moments are unequal; viz, Δ*E*_Z_=−(***μ***^c^−***μ***^v^)·**B**. In the following, we consider strictly out-of-plane fields, 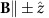.

Figure 1b depicts the field-dependent energy shifts of the conduction and valence bands in the *K* valley (*σ*^+^ polarized light), for both positive and negative fields. The various contributions to the total Zeeman shift in the monolayer TMDs have been discussed in several recent reports^3^,^12^,^13^,^14^,^15^,^44^, which we summarize as follows. In general, the total magnetic moment ***μ*** of any given conduction or valence band in the *K* or *K*′ valley contains contributions from three sources: spin (*μ*_s_); atomic orbital (*μ*_l_); and the valley orbital magnetic moment that is associated with the Berry curvature (*μ*_k_). Note that the latter two have been referred to as ‘intracellular' and ‘intercellular' contributions to the orbital magnetic moment, respectively^12^,^13^. The spin contribution to the exciton Zeeman shift Δ*E*_Z_ is expected to be zero, since the optically allowed transitions couple conduction and valence bands having the same spin 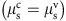. In contrast, the conduction and valence bands are comprised of entirely different atomic orbitals: the 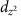 orbitals of the conduction bands have azimuthal orbital angular momentum *l*_*z*_=0 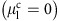, while the hybridized 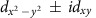 orbitals that comprise the valence bands have *l*_*z*_=±2*ħ* 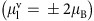 in the *K* and *K*′ valleys, respectively. This contribution is expected to generate a Zeeman shift of the *K* and *K*′ exciton of 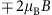, respectively, and therefore, a total exciton splitting of −4*μ*_B_*B*. Finally, the valley orbital (Berry curvature) contributions to the conduction and valence band moments are 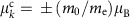 and 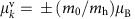 in the *K* and *K*′ valleys, respectively. In a simple two-band tight-binding model where *m*_e_=*m*_h_, then 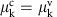 and shifts due to the valley orbital magnetic moment do not appear in Δ*E*_Z_. In more general models^45^ where *m*_e_≠*m*_h_, these Berry curvature contributions may play a role and cause a deviation of the exciton Zeeman splitting away from −4*μ*_B_.

To selectively probe the *K* and *K*′ transitions in our magneto-reflectivity experiments, we typically fixed the handedness of the circular polarizer to *σ*^+^, and pulsed the magnet in the positive (+65 T) and then the negative (−65 T) field direction. The latter case is exactly equivalent (by time-reversal symmetry) to measuring the *σ*^−^ optical transitions in positive field (we also explicitly verified this by changing the circular polarizer). Sign conventions were confirmed via magneto-reflectance from a diluted magnetic semiconductor (Zn_0.92_Mn_0.08_Se)^46^.

### Valley Zeeman effect in monolayer WS_2_

**Figure 2:**
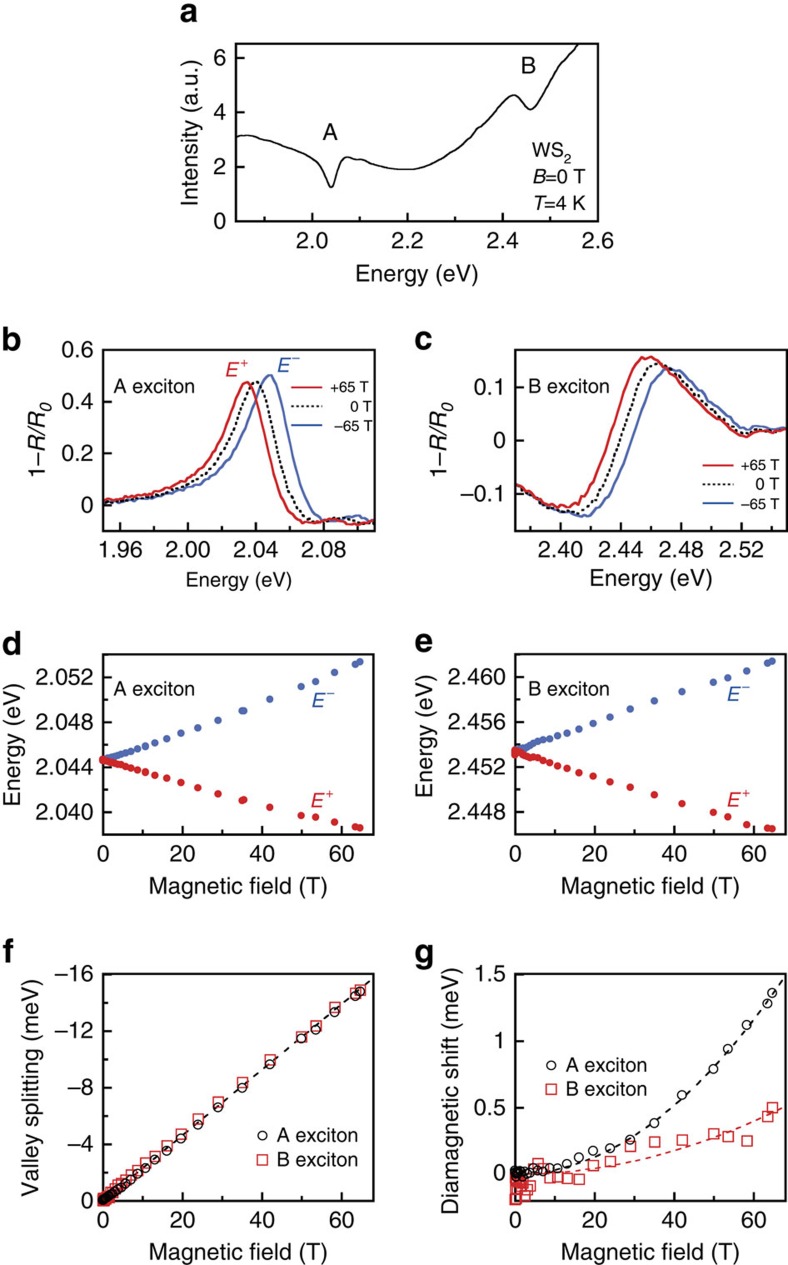
Valley Zeeman effect and diamagnetic shift in monolayer WS_2_. (**a**) Reflection spectrum of monolayer WS_2_ at *B*=0 T and *T*=4 K. The A and B exciton resonances are labelled. (**b**) Normalized reflection spectra (1−*R*/*R*_0_) at the A exciton resonance using *σ*^+^ polarized light. The dashed black trace was acquired at *B*=0 T. The red trace was acquired at +65 T, and corresponds to the *σ*^+^ transition in the *K* valley. The blue trace was acquired at −65 T, which is equivalent (by time-reversal symmetry) to the *σ*^−^ transition in the *K*′ valley at +65 T. The valley Zeeman splitting between these two peaks is clearly resolved. (**c**) Similar reflection spectra and valley Zeeman splitting of the B exciton. (**d**) Energies (*E*^+^ and *E*^−^) of the field-split A exciton versus magnetic field. (**e**) Same, but for the B exciton. (**f**) The measured valley Zeeman splitting (*E*^+^−*E*^−^) versus magnetic field, for both A and B excitons. The dotted line corresponds to a splitting of −4*μ*_B_ (−232 μeV T^−1^). (**g**) The average energy, (*E*^+^ +*E*^−^)/2, for both the A and B excitons (the zero-field offset has been subtracted). A small quadratic diamagnetic shift is revealed. The dotted lines show quadratic fits to the data (Δ*E*_dia_=*σB*^2^), where *σ* is the diamagnetic shift coefficient. We find that *σ*_A_=0.32±0.02 μeV T^−2^ and *σ*_B_=0.11±0.02 μeV T^−2^ for the A and B excitons, respectively.

Figure 2a shows the reflection spectrum (raw data) from monolayer WS_2_ at 4 K. Both the A and B exciton transitions are clearly visible and are superimposed on a smoothly varying background. Figure 2b shows the well-resolved Zeeman splitting of the A exciton in WS_2_ at the maximum ±65 T applied magnetic field. Red, blue and (dashed) black curves show the normalized reflection spectra, 1−*R*/*R*_0_ (where *R*_0_ is a smooth background), at +65, −65 and 0 T respectively. A valley splitting of ∼15 meV, analysed in detail below, is observed. Moreover, because these measurements are based on magneto-reflectance spectroscopy (rather than photoluminescence), the valley splitting of the higher-energy B exciton in WS_2_ can also be observed, as shown in Fig. 2c. For both the A and B excitons, the energy of the exciton transition in positive magnetic fields (hereinafter called *E*^+^) shifts to lower energy, while the exciton energy in negative fields (*E*^−^) shifts to higher energy, as labelled.

The exciton resonances were fit using complex (absorptive+dispersive) Lorentzian lineshapes to extract the transition energy. The field-dependent energies of the split peaks in monolayer WS_2_, *E*^+^(*B*) and *E*^−^(*B*), are shown in Fig. 2d,e for the A and B excitons, respectively. The splitting between the two valleys, *E*^+^−*E*^−^, is shown in Fig. 2f for both the A and B excitons. The measured valley Zeeman splitting is negative, and increases in magnitude linearly with applied field, with nearly identical rates of −228±2 μeV T^−1^ for the A exciton and −231±2 μeV T^−1^ for the B exciton. These values correspond to Landé *g*-factors of −3.94±0.04 and −3.99±0.04, respectively, thereby quantifying the valley Zeeman effect in the monolayer transition-metal disulphides, and also providing a measurement of the B exciton valley splitting in monolayer TMD materials.

The A exciton valley splitting that we measure in monolayer WS_2_ is quite close to that reported recently from magneto-photoluminescence studies of its diselenide counterpart, monolayer WSe_2_ (refs 13, 16). For comparison, reported *g*-factors for all the monolayer TMDs are shown in Table 1. As discussed above, our measured values of *g*≃−4 agree surprisingly well with a simple two-band tight-binding model, wherein *m*_e_=*m*_h_ and valley moment (Berry curvature) contributions to the exciton magnetic moment cancel out, so that the exciton Zeeman shifts derive solely from atomic orbital magnetic moments of the valence bands. However, Berry curvature contributions to the Zeeman splitting are expected in more general models^45^ where *m*_e_≠*m*_h_. Deviations away from *g*=−4, observed, for example, in refs 13, 14, have been explained along these lines (although, note that for tightly bound excitons, the total valley moment contribution can vary significantly in magnitude and sign, because this orbital moment must be averaged over a substantial portion of the Brillouin zone^13^).

In view of the above, it is therefore particularly noteworthy that we also measure *g*≃−4 for the B exciton in monolayer WS_2_, despite the fact that its reduced mass almost certainly differs from that of the A exciton, as shown below from direct measurements of the diamagnetic shift (that is, *m*_h_ cannot equal *m*_e_ for both spin-up and spin-down valence bands). Note that early studies of bulk MoS_2_ (refs 47, 48, 49) also indicate that the B exciton mass significantly exceeds that of the A exciton. This suggests that contributions to the orbital moment from Berry curvature effects, expected when *m*_e_≠*m*_h_, may not play a significant role in determining the measured exciton magnetic moment and the valley Zeeman effect.

### Non-local dielectric screening in monolayer TMDs

In addition to the reduced mass *m*_r_, the characteristic size of the A and B excitons in monolayer TMDs is an essential parameter for determining material and optical properties. This is especially relevant because of non-local dielectric screening in these and other 2D materials, which fundamentally modifies the functional form of the attractive potential *V*(*r*) between electrons and holes^23^,^36^,^37^. Rather than a conventional Coulomb potential, *V*(*r*) is believed to assume the following form:


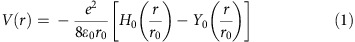


where *H*_0_ and *Y*_0_ are the Struve function and Bessel function of the second kind, respectively, and the characteristic screening length 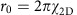, where *χ*_2D_ is the 2D polarizability of the monolayer material^23^,^37^. This potential follows a 1/*r* Coulomb-like potential for large electron–hole separations 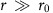, but diverges weakly as log(*r*) for small separations 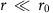, leading to a markedly different Rydberg series of exciton states with modified wavefunctions and binding energies that cannot be described within a hydrogen-like model^23^,^24^,^25^,^27^.

### Diamagnetic shifts in monolayer WS_2_

To this end, the use of very large 65 T magnetic fields allows us to measure the small diamagnetic shifts of excitons in monolayer TMDs so that the characteristic size of their wavefunctions can be directly inferred. In general^20^,^21^,^22^, an exciton diamagnetic shift Δ*E*_dia_ is expressed as


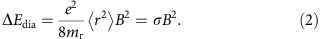


Here *σ* is the diamagnetic shift coefficient, *m*_r_ is the in-plane reduced mass, *r* is a radial coordinate in a plane perpendicular to the applied magnetic field *B* (here for 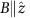, *r* is in the monolayer plane) and 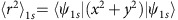 is the expectation value of *r*^2^ over the 1*s* exciton wavefunction 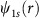. Equation (2) applies in the ‘low-field' limit where the characteristic cyclotron energies *ħω*_c_ (and also Δ*E*_dia_) are less than the exciton binding energy, which is the case for excitons in TMDs even at ±65 T. Given *m*_r_, *σ* can then be used to determine the r.m.s. radius of the 1*s* exciton in the monolayer plane, *r*_1_:


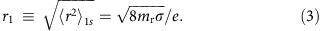


This definition is entirely general and independent of *V*(*r*). (Note that for a standard Coulomb potential *V*(*r*)=−*e*^2^/(4*πɛ*_*r*_*ɛ*_0_*r*) in two dimensions, 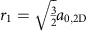, where *a*_0,2D_=2*πɛ*_*r*_*ɛ*_0_*ħ*^2^/*m*_r_*e*^2^ is the classic Bohr radius for hydrogenic 2D excitons.)

Exciton diamagnetic shifts have eluded detection in recent magneto-photoluminescence studies of monolayer MoSe_2_ and WSe_2_ (refs 12, 13, 14, 15, 16), likely due to the limited field range employed (|*B*|<10 T). Here the diamagnetic shift of the A exciton in monolayer WS_2_ can be seen in 65 T fields via the slight positive curvature of both *E*^+^(*B*) and *E*^−^(*B*) in Fig. 2d. To directly reveal Δ*E*_dia_, Fig. 2g shows the average exciton energy, (*E*^+^+*E*^−^)/2. Overall quadratic shifts are indeed observed, indicating diamagnetic coefficients *σ*_A_=0.32±0.02 μeV T^−2^ for the A exciton and a smaller value of *σ*_B_=0.11±0.02 μeV T^−2^ for the B exciton. These measurements were repeated on five different regions of the monolayer WS_2_ sample, with similar results.

### Exciton radii and binding energies

Importantly, knowledge of *σ* constrains not only the r.m.s. exciton radius *r*_1_ (if the mass is known) but also the exciton binding energy if the potential *V*(*r*) is known. Theoretical estimates^3^,^23^,^31^,^32^ for the A exciton reduced mass in monolayer WS_2_ range from 0.15 to 0.22*m*_0_, from which we can then directly calculate *r*_1,A_=1.48–1.79 nm via equation (3). These values are in reasonable agreement with recent *ab initio* calculations of the 1*s* exciton wavefunction in monolayer WS_2_ (ref. 25), and further support a picture of 2D Wannier-type excitons with lateral extent larger than the monolayer thickness (0.6 nm) and spanning several in-plane lattice constants.

**Figure 3:**
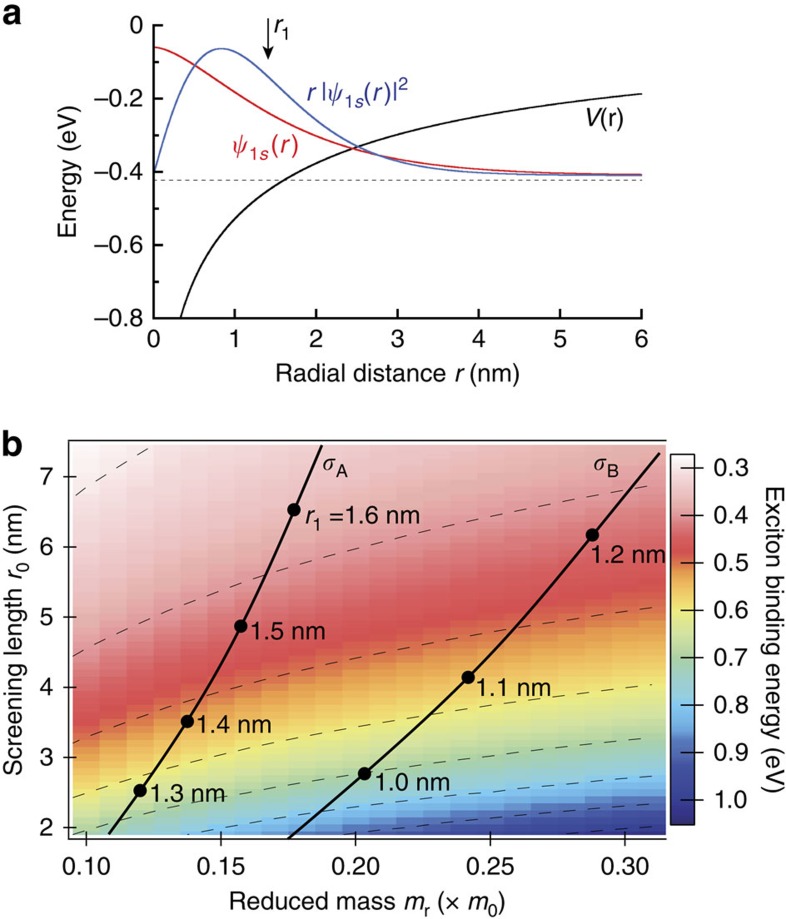
Constraining exciton binding energies via the diamagnetic shift. (**a**) A plot of the (non-hydrogenic) 1*s* A exciton wavefunction in monolayer WS_2_, 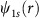, computed by numerically solving Schrödinger's equation using a reduced mass *m*_r_=0.16 and the non-local dielectric screening potential *V*(*r*) defined in equation (1). The screening length *r*_0_ was adjusted to give 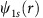 such that the r.m.s. exciton radius 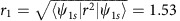 nm, which is the value calculated from the measured diamagnetic shift *σ*_A_=0.32 μeV T^−2^. (Note that *r*_1_ is an r.m.s. value, and does not correspond to the peak of the 2D radial probability density 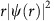.) (**b**) Colour surface plot of the calculated exciton binding energy, using *V*(*r*) from equation (1), over a range of reduced mass *m*_r_ and screening length *r*_0_. Dashed lines show contours of constant binding energy. Solid lines indicate contours of constant diamagnetic shift corresponding to those measured in Fig. 2g for the A and B excitons in monolayer WS_2_. The calculated r.m.s. exciton radius *r*_1_ is indicated at various points along the contours.

Moreover, *σ*, *m*_r_ and *r*_1_ can then be used to calculate the A exciton wavefunction 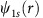 and its binding energy, by numerically solving the 2D Schrödinger equation for describing the relative motion of electrons and holes using the potential *V*(*r*) as defined in equation (1), and taking the screening length *r*_0_ as an adjustable parameter to converge on a solution for 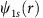 that has the correct r.m.s. radius *r*_1_. For example, using *m*_r,A_=0.16*m*_0_ for the A exciton in WS_2_, and using the measured diamagnetic shift *σ*_A_, we find that *r*_1,A_=1.53 nm via equation (3). A wavefunction 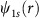 with this r.m.s. radius, shown explicitly in Fig. 3a, is calculated if (and only if) the screening length *r*_0_=5.3 nm, and the binding energy of this state is 410 meV. For comparison, this inferred screening length is somewhat larger than expected for a suspended WS_2_ monolayer (where *r*_0_=2*πχ*_2D_=3.8 nm; ref. 23), but is less than the value of 7.5 nm used recently by Chernikov^24^ to fit a non-hydrogenic Rydberg series of excitons in WS_2_ from reflectivity data. Similarly, the 410 meV exciton binding energy that we estimate exceeds the value inferred by Chernikov (320 meV), but is less than the 700–830 meV binding energies extracted from two-photon excitation studies^25^,^26^ and reflectivity/absorption studies^28^ of monolayer WS_2_. We emphasize, however, that the exciton wavefunctions and binding energies that we calculate necessarily depend on the reduced mass *m*_r_ and the exact form of the potential *V*(*r*), which is sensitive to the details of the dielectric environment and choice of substrate material^33^,^50^.

More generally, Fig. 3b shows a colour-coded surface plot of the exciton binding energy, calculated within the framework of the non-local dielectric screening potential *V*(*r*) defined in equation (1), over a range of reduced masses *m*_r_ and effective dielectric screening lengths *r*_0_. At each point, the 1*s* exciton wavefunction 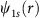, its binding energy, and its r.m.s. radius *r*_1_ were calculated, from which we computed the expected diamagnetic shift coefficient 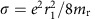. Importantly, the solid lines on the plot indicate the contours of constant diamagnetic shift that correspond to our experimentally measured values *σ*_A_ and *σ*_B_. At intervals along these contours, *r*_1_ is indicated. From this plot, it can be immediately seen that over the range of theoretically calculated masses (*m*_r,A_=0.15–0.22*m*_0_), excitons having the appropriate size to give the measured diamagnetic shift *σ*_A_ (that is, those lying along the *σ*_A_ contour) have binding energies in the range of 480–260 meV. Within this model, excitons with even larger binding energies (but still constrained to exhibit the correct diamagnetic shift) are anticipated if the reduced mass *m*_r_ is lighter and the effective screening length *r*_0_ is smaller.

In addition, Fig. 3b also allows us to estimate the mass, binding energy and spatial extent of the B exciton in monolayer WS_2_, for which a smaller diamagnetic shift of *σ*_B_=0.11 μeV T^−2^ was measured. Assuming that the local dielectric environment is similar for A and B excitons (that is, *r*_0_ is unchanged), then parameters for the B exciton lie at a point on the *σ*_B_ contour that is directly to the right of those on the *σ*_A_ contour. Thus, if *m*_r,A_=0.16*m*_0_ and *r*_1,A_=1.53 nm as discussed above, then the B exciton reduced mass is *m*_r,B_=0.27*m*_0_, its r.m.s. radius is *r*_1,B_=1.16 nm and its binding energy is 470 meV. These values are qualitatively consistent with trends identified in early optical studies of bulk MoS_2_ crystals^48^,^49^, in which B exciton masses and binding energies were found to exceed those of A excitons. These results highlight a further interesting consequence of the potential *V*(*r*), which is that exciton binding energies scale only weakly and nonlinearly with *m*_r_, in contrast to the case for hydrogenic potentials.

### Zeeman splitting and diamagnetic shifts in bulk WS_2_

**Figure 4:**
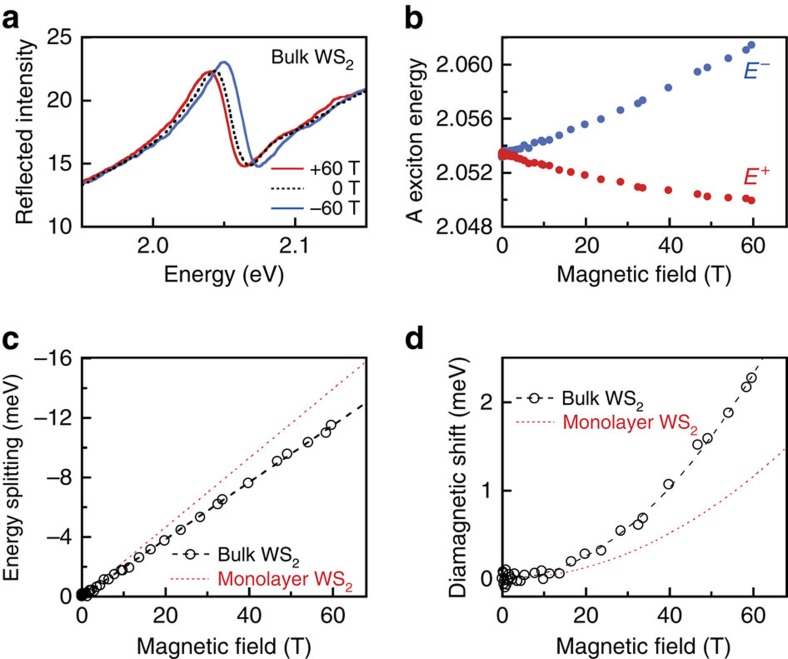
Zeeman splitting and diamagnetic shift in bulk WS_2_. (**a**) Intensity of reflected *σ*^+^ light from the A exciton in bulk WS_2_ at 4 K, using *B*=0, +60 and −60 T. (**b**) Energies of the Zeeman-split exciton transitions, *E*^+^ and *E*^−^. (**c**) The measured exciton splitting (*E*^+^−*E*^−^), corresponding to *g*=−3.33. For comparison, the red line shows the valley Zeeman splitting measured in monolayer WS_2_ (*cf.* Fig. 2f). (**d**) The average energy (*E*^+^+*E*^−^)/2 showing a diamagnetic shift (0.64 μeV T^−2^) that is twice as large as that measured in monolayer WS_2_ (red line; *cf.* Fig. 2g).

For direct comparison with monolayer WS_2_, circularly polarized magneto-reflectance measurements were also performed on the exfoliated surface of a bulk WS_2_ crystal (grown by chemical vapour transport at the Tennessee Crystal Center). Figure 4a shows the well-known^51^ A exciton resonance in bulk WS_2_, which arises from the lowest-energy direct optical transition that is located at the *K* points of the Brillouin zone^52^ (this transition, with only slight modification in energy, eventually becomes the lowest overall transition when WS_2_ is thinned to a single monolayer and becomes a direct-gap semiconductor^1^,^2^). At low temperatures and in ±60 T magnetic fields, the Zeeman splitting of the bulk A exciton is readily resolved (Fig. 4a–c) and is found to increase linearly with field at a rate of −193 μeV T^−1^ (*g*=−3.33). This value is in very close agreement with early magnetic circular dichroism measurements of *g*-factors in bulk WS_2_ (ref. 53), wherein it was suggested that deviations from *g*=−4 arise from the crystal-field mixing of *p*-type chalcogen atomic orbitals into the predominantly *d*-type character of the conduction and valence bands. Within this context, the value of *g*≃−4 that we measured in monolayer WS_2_ (Fig. 2f) may suggest that such mixing effects, if present, may be suppressed in atomically thin WS_2_.

In addition, Fig. 4d shows that the measured diamagnetic shift of the A exciton in bulk WS_2_ is 0.64 μeV T^−2^, which is twice as large as in monolayer WS_2_. Assuming an in-plane reduced mass of *m*_r_=0.21*m*_0_ in bulk WS_2_ (ref. 51), we calculate via equation (3) an in-plane r.m.s. radius of *r*_1_=2.48 nm for the bulk A exciton, which is substantially larger than that inferred for monolayer WS_2_. This large r.m.s. radius indicates an effective dielectric screening constant *ɛ*_r_≃7.0, in agreement with early work^51^, and from which the A exciton binding energy in bulk WS_2_ can be estimated via the standard hydrogenic formulation, 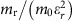 × 13.6 eV=58 meV. This value agrees extremely well with early work on bulk WS_2_ (ref. 51), and is close to that found in other bulk TMDs^54^. Therefore, we find that the binding energy of the A exciton in WS_2_ increases by approximately a factor of 7 on reducing the dimensionality of the host crystal from three-dimensional to 2D. Note, however, that these estimates depend on the assumed value of the reduced mass *m*_r_, which has not yet been measured independently by, for example, cyclotron resonance studies.

### Valley Zeeman effect in monolayer MoS_2_

**Figure 5:**
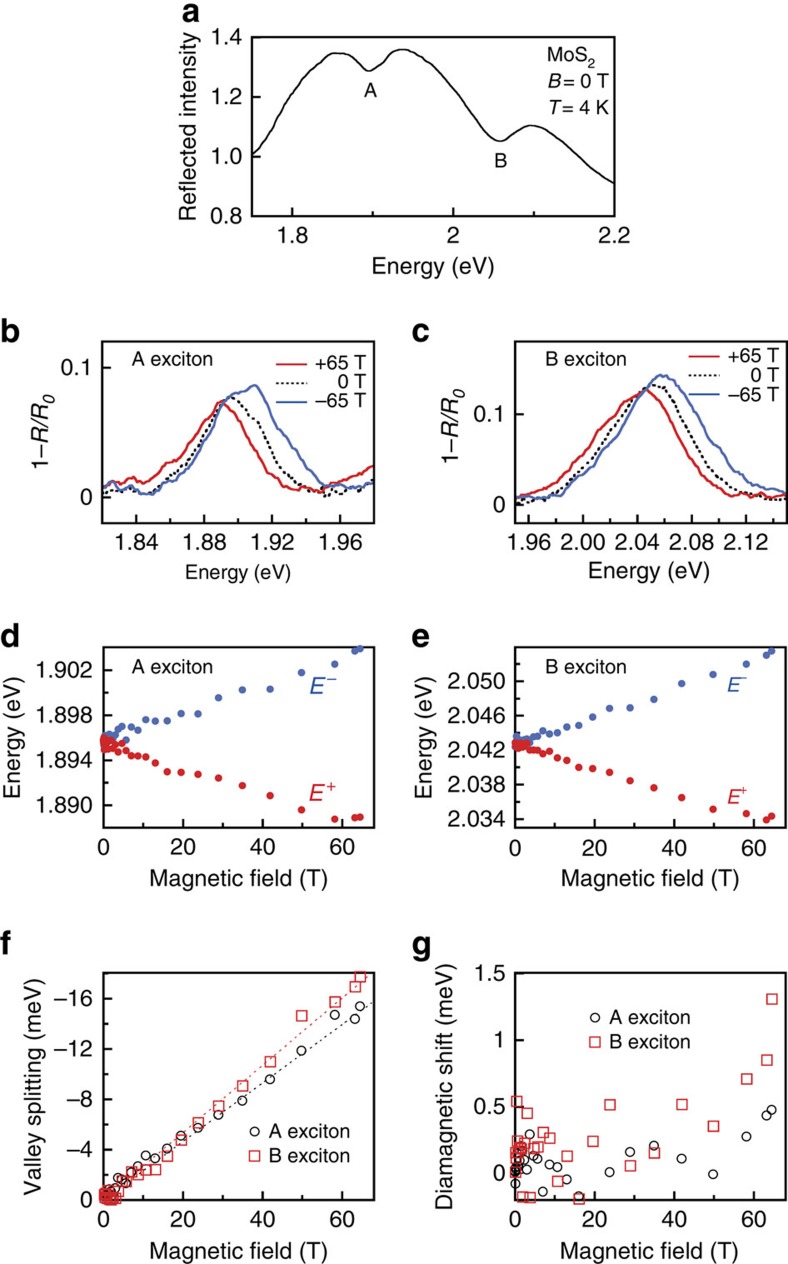
Valley Zeeman effect in monolayer MoS_2_. (**a**) Reflection spectrum of monolayer MoS_2_ at *B*=0 T and *T*=4 K. The A and B exciton resonances are labelled. (**b**,**c**) Normalized reflection spectra (1−*R*/*R*_0_) at the A and B exciton resonances at *B*=0, +65 and −65 T. (**d**) Energies of the field-split A exciton transition. (**e**) Same, but for the B exciton transition. (**f**) The measured valley splitting (*E*^+^−*E*^−^) versus the magnetic field, for both A and B excitons. (**g**) The average energy of the two valley-split resonances, (*E*^+^+*E*^−^)/2, for both A and B excitons. Increased scatter in the data from this MoS_2_ sample precludes any clear identification of the diamagnetic shift.

To complete this study of the monolayer transition-metal disulphides, we also performed high-field magneto-reflectance studies on large-area samples of monolayer MoS_2_ (Fig. 5). The A and B exciton linewidths are broader and the optical reflection contrast is lower than for monolayer WS_2_ (Fig. 5a). Nonetheless, a clear valley Zeeman splitting of both excitons is observed (Fig. 5b,c). The energies of the field-split exciton peaks are shown in Fig. 5d,e for the A and B excitons, respectively. Although the reduced signals and broader features lead to increased scatter in the fitted data, Fig. 5f shows that the measured valley splitting of the A and B excitons in MoS_2_ increases approximately linearly with field at rates of −233±10 and −270±10 μeV T^−1^, corresponding to *g*≃−4.0±0.2 and −4.65±0.17, respectively. For the A exciton, this value is very close to those inferred from low-field magneto-photoluminescence studies^12^,^15^,^16^ of their diselenide counterpart, monolayer MoSe_2_ (Table 1). As discussed above for the case of monolayer WS_2_, a *g*-factor of −4 for the A exciton agrees surprisingly well with expectations from a simple two-band tight-binding picture, and suggests that the valley Zeeman effect in MoS_2_, much like MoSe_2_, is largely uninfluenced by contributions from the valley orbital (Berry curvature) magnetic moment. We note, however, that the measured valley *g*-factor is somewhat larger for the B exciton in monolayer MoS_2_. Unfortunately, the reduced signal levels from these monolayer MoS_2_ samples led to correspondingly increased scatter in the fitted exciton energies, precluding an accurate determination of exciton diamagnetic shifts in monolayer MoS_2_ (Fig. 5g).

## Discussion

In summary, we have presented a comprehensive study of valley Zeeman effect and diamagnetic shifts of excitons in the archetypal monolayer transition-metal disulphides WS_2_ and MoS_2_. Valley *g*-factors of the A excitons are approximately −4, which are similar to those obtained from transition-metal diselenides. Unexpectedly, the heavier B exciton in monolayer WS_2_ also exhibits *g*≃−4, suggesting that the valley Zeeman effect is largely unaffected by the exciton reduced mass. The very large magnetic fields used in these studies also allowed initial measurements of the exciton diamagnetic shifts in a monolayer TMD—specifically, WS_2_—from which r.m.s. exciton radii were directly computed (*r*_1_=1.53 and 1.16 nm for the A and B excitons, respectively). Within a picture of non-local dielectric screening in these 2D semiconductors, these measurements of diamagnetic shifts allowed us to constrain estimates of the exciton binding energies, which we calculate (using a reduced A exciton mass of 0.16*m*_0_) to be 410 and 470 meV for the A and B excitons, respectively, in monolayer WS_2_. These studies highlight the utility of very large magnetic fields for characterizing new 2D material systems.

## Additional information

**How to cite this article:** Stier, A. V. *et al*. Exciton diamagnetic shifts and valley Zeeman effects in monolayer WS_2_ and MoS_2_ to 65 Tesla. *Nat. Commun.* 7:10643 doi: 10.1038/ncomms10643 (2016).

## Supplementary Material

Supplementary Information Supplementary Figures 1-5, Supplementary Notes and Supplementary References

## Figures and Tables

**Table 1:** Summary of effective *g*-factors and diamagnetic shifts in monolayer TMDs.

Material	A exciton *g*-factor	B exciton *g*-factor	*σ*_A_ (μeV T^−2^)	*σ*_B_ (μeV T^−2^)
Monolayer WS_2_	**−3.94**±**0.04**		**0.32**±**0.02**	
	**−3.33**±**0.04** (bulk)	**−3.99**±**0.04**	**0.64**±**0.02** (bulk)	**0.11**±**0.02**
	−3.2±0.3 (bulk)^53^			
Monolayer MoS_2_	**−4.0**±**0.2**			
	−4.6±0.08 (bulk)^55^	**−4.65**±**0.17**	—	—
	−3.1±0.1 (bulk)^53^			
Monolayer WSe_2_	−3.7±0.2 (ref. 16)	—	—	—
	−4.37±0.15 (ref. 13)			
	−1.57 to −2.86 (ref. 14)			
	−4±0.5 (ref. 17)			
Monolayer MoSe_2_	−3.8±0.2 (refs 12, 16)	—	—	
	−4.1±0.2 (ref. 15)			

Experimentally measured exciton *g*-factors corresponding to the valley Zeeman effect, and diamagnetic shift coefficients *σ*, in monolayer transition-metal dichalcogenide materials. Measurements from this work are indicated in boldface. For comparison, also included are selected measurements from bulk crystals.
