# Supplementary material for: Exciton diamagnetic shifts and valley Zeeman effects in monolayer WS2 and MoS2 to 65 Tesla
Source: Nat Commun. 2016 Feb 9;7:10643. doi: 10.1038/ncomms10643 (PMC4748133; doi:10.1038/ncomms10643)
Supplement: Supplementary Information — Supplementary Figures 1-5, Supplementary Notes and Supplementary References [file ncomms10643-s1.pdf]

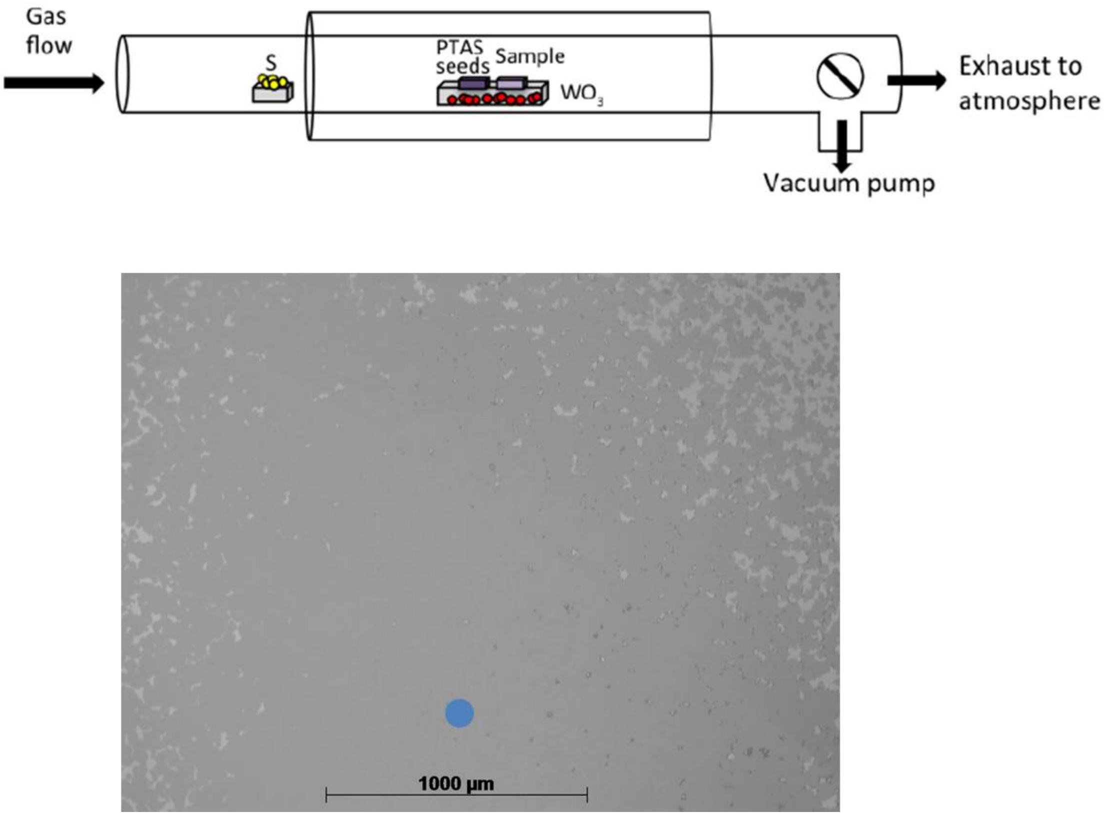

Supplementary Figure 1: CVD growth of large-area monolayer WS_2 (a) Schematic of the CVD setup. A similar setup was utilized for the MoS_2 growth. (b) Large scale microscope optical image of the WS_2 film on the Si/SiO_2 substrate. The blue circle indicates the typical spot size (~100 µm) of the focused white light used in the pulsed-field reflection experiments.

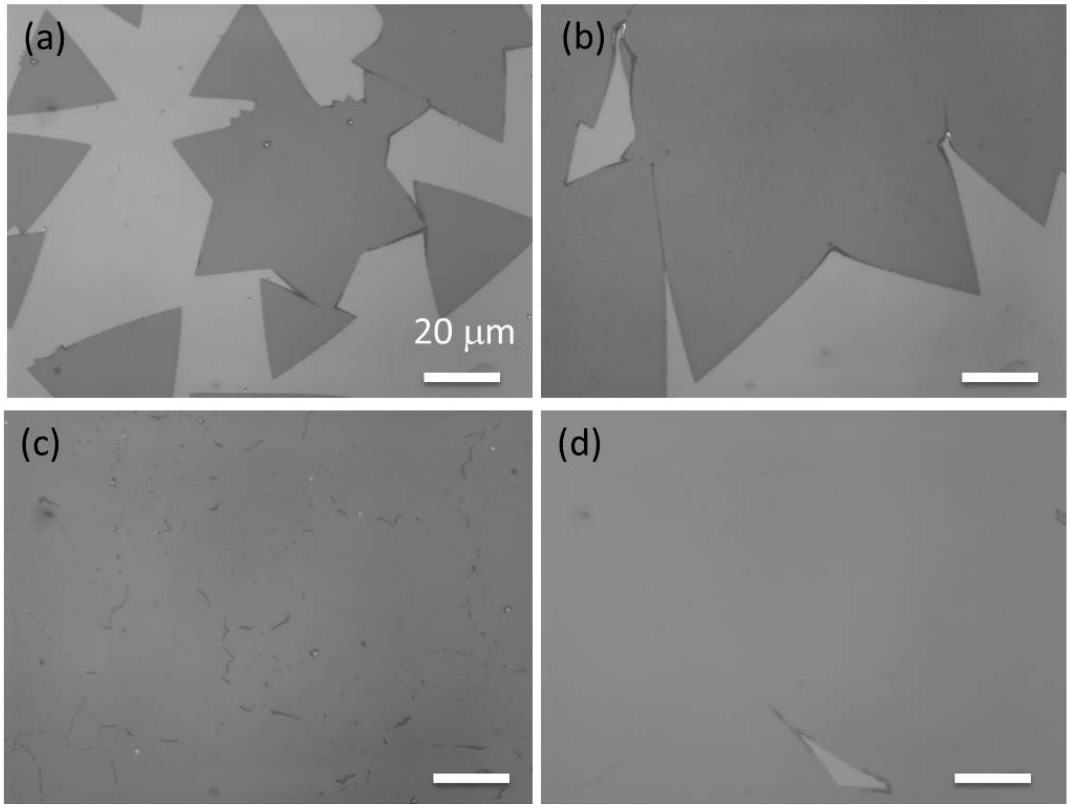

Supplementary Figure 2: WS_2 high resolution optical images (a-d) High magnification optical images of WS_2 on Si/SiO_2 obtained from multiple samples and various regions. The border region of continuous film growth is shown in (a) and (b). Multilayer growth is found on the edges of monolayer islands. (c-d) Regions of continuous monolayer WS_2 film growth (97% and 99% coverage, respectively), showing faint multilayer streaks.

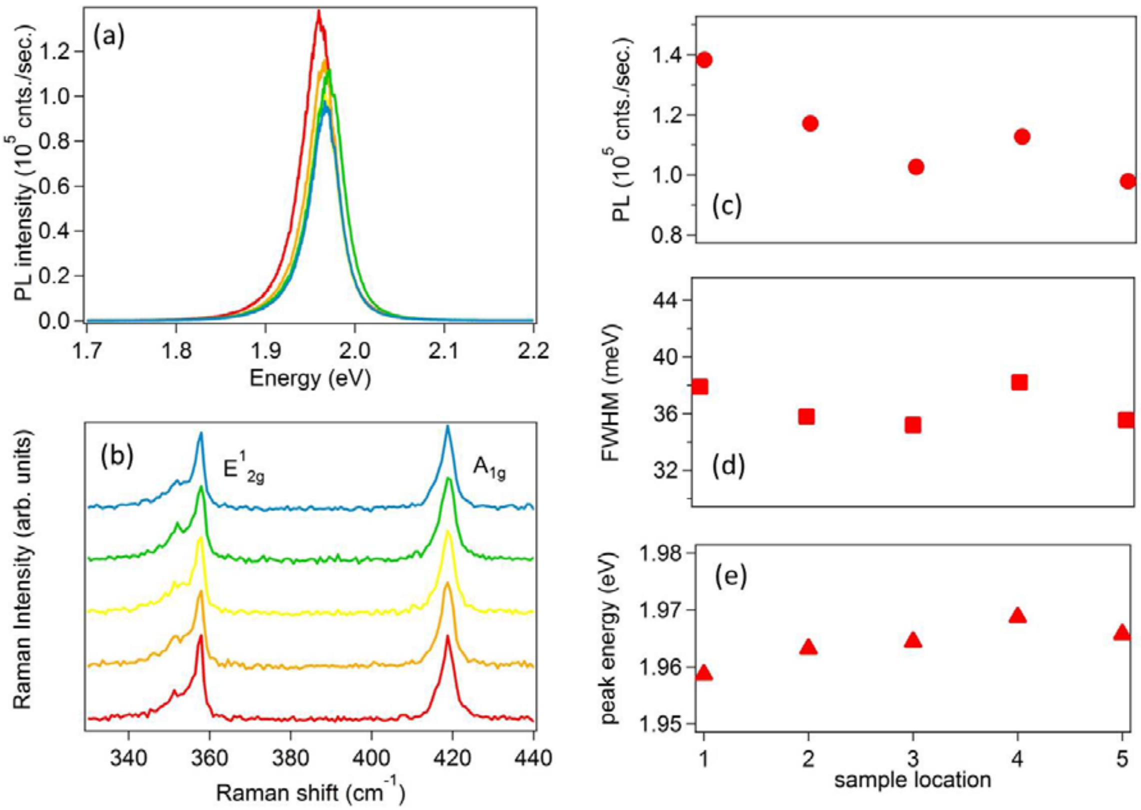

Supplementary Figure 3: PL and Raman spectra of WS_2 (a) Photoluminescence of the WS_2 film at 5 random places across the film (minimum 500 µm apart). (b) Raman spectra at the same spots as the PL. (c-e) Deduced parameters of Gaussian fits to the PL data in (a).

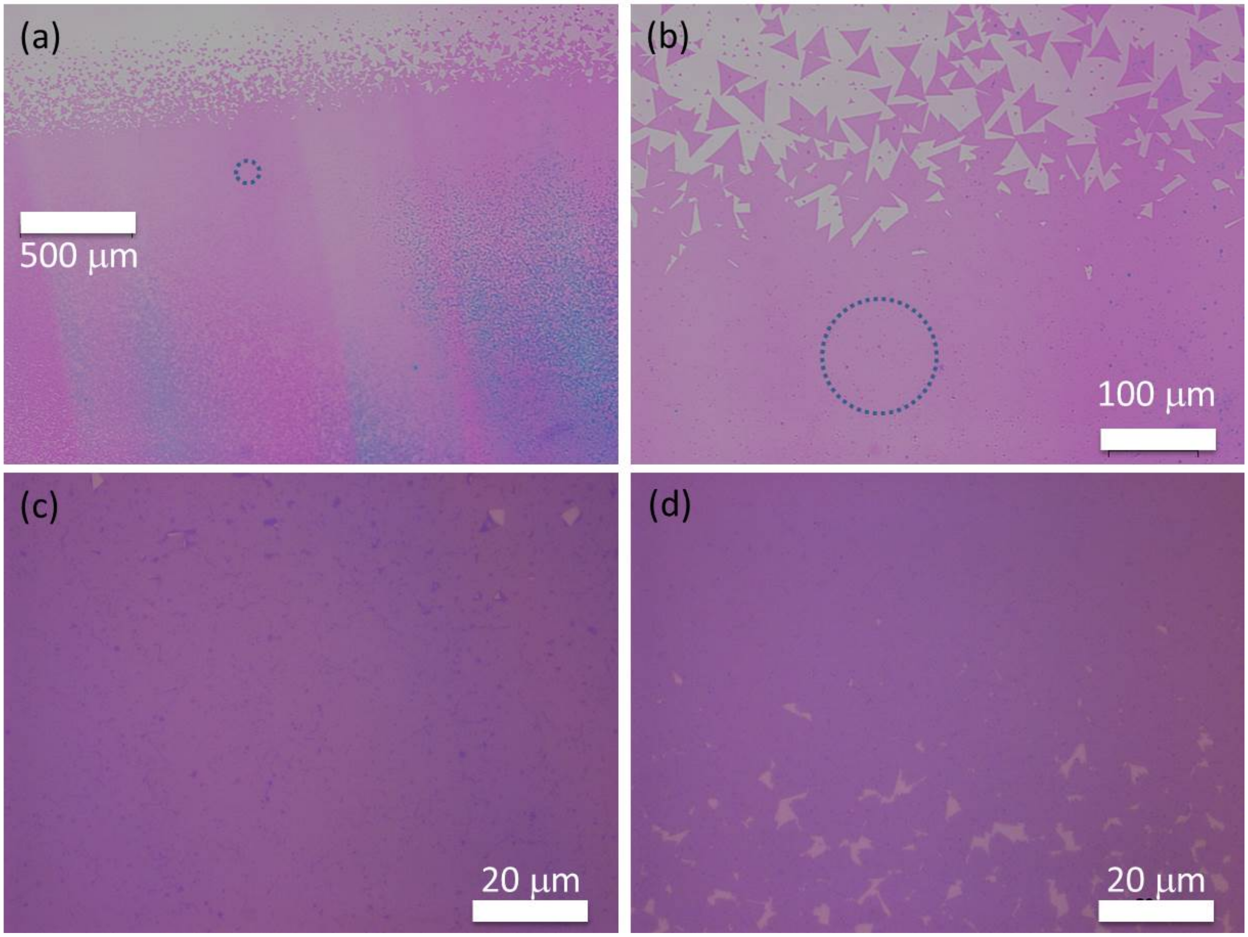

Supplementary Figure 4: MoS_2 optical images: (a) 5x and (b) 20x optical microscope images of a MoS_2 film on a Si/SiO_2 substrate. The dotted circles in (a-b) indicate the spot size of the focused white light (100 μm). (c-d) Detailed 100x images of the MoS_2 monolayer area.

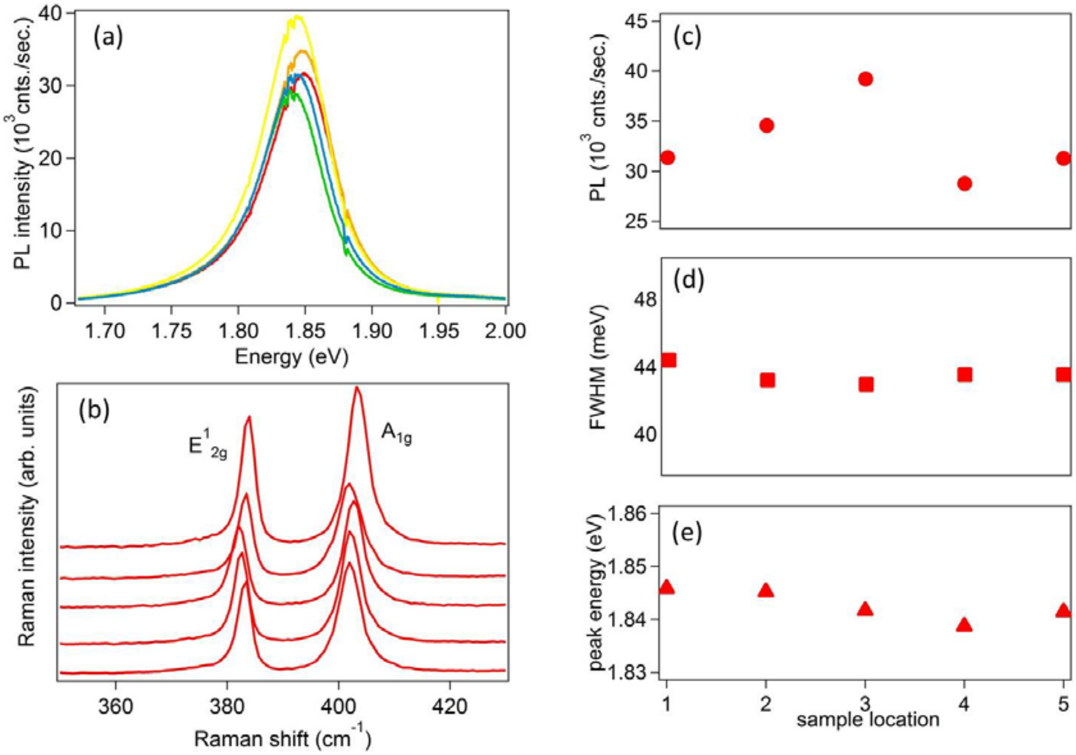

Supplementary Figure 5: PL and Raman spectra of MoS_2 (a) PL of the MoS_2 film at 5 random positions spanning the film (minimum 500 µm apart). (b) Raman spectra of the MoS_2 film at 5 random positions of monolayer coverage. (c-e) Parameters of fits to the PL data in (a).

Supplementary Note 1: WS_2 and MoS_2 monolayer CVD growth.

Complete details of the sample growth and characterization of large-area WS_2 monolayers can be found in Reference [1]. The samples used in this high-field magneto-reflectance study follow "recipe C" in [1]. Supplementary Figure 1 shows a schematic of the CVD growth system. Synthesis of monolayer transition metal dichalcogenides (TMDs) is performed at ambient pressure in a 2-inch diameter quartz tube furnace. Si/SiO_2 (275 nm) wafers are used as substrate material. Prior to growth, all substrates undergo a standard cleaning procedure consisting of (i) ultrasonic bath in acetone (ii) ultrasonic bath in isopropyl alcohol (iii) submersion in Piranha etch (3:1 mixture of H_2SO_2:H_2O_2) for 2 hours and (iv) thorough rinsing in de-ionized water. At the center of the furnace, a quartz boat containing 1g of WO_3 powder (45 mg of MoO_3) for synthesis of WS_2 (MoS_2) is positioned. Two Si/SiO_2 substrates are placed face-down, directly above the oxide precursor. The upstream wafer contains perylene-3,4,9,10-tetracarboxylic acid tetrapotassium salt (PTAS) seeding molecules, while the downstream substrate is untreated. The hexagonal PTAS molecules are carried downstream to the untreated substrate and promote lateral growth of the TMD materials [2]. A separate quartz boat containing sulfur powder is placed upstream, outside the furnace-heating zone.

Monolayer MoS_2 is grown under continuous flow of argon (100 sccm). The furnace temperature is ramped to 625 °C, held constant for 10 minutes, and then allowed to cool. To achieve monolayer WS_2 growth, pure argon (100 sccm) is used as the furnace heats to the target temperature. Upon reaching 825 °C, 10 sccm H_2 is added to the argon flow and maintained throughout the 10 minute growth time and subsequent cooling.

A large-area optical image of the film is shown in Supplementary Figure 1. The blue circle indicates the spot size of the focused white light on the sample. As can be seen, the spot size is smaller than the relevant spatial scales for uniform monolayer coverage of the WS_2 film.

Supplementary Note 2: Optical characterization of the monolayer WS_2 film

High magnification optical images of various samples and regions on the film are shown in Supplementary Figure 2. Each panel displays an area of 125 µm x 95 µm. Regions of the bare substrate, monolayer WS_2, and multilayer WS_2 growth are clearly discernible based on the optical contrast (the darkest regions depict multilayer WS_2). Multilayer growth typically occurs at the edges of otherwise monolayer triangles in the border region of continuous monolayer films (see Supplementary Figure 2 a-b). Similar multilayer border regions can be observed in areas of continuous film coverage (Supplementary Figures 2c-d). We speculate that these multilayer streaks indicate a boundary line where two growth islands have merged. The monolayer coverage, as determined from software image analysis of the optical contrast, is routinely better than 97%. For example, Supplementary Figure 2c shows 97% monolayer coverage and Supplementary Figure 2 (d) shows 99% monolayer coverage.

Supplementary Note 3: Photoluminescence and Raman spectroscopy of the WS_2 monolayers

Photoluminescence (PL) and Raman scattering studies of the WS_2 films were performed at room temperature in air using a 488 nm laser with intensity below 200 µW for a <1µm diameter spot size to prevent sample damage. Supplementary Figure 3 shows the PL and Raman spectra at 5 randomly-selected sites on the film. Sample quality can be gauged by the full width at half maximum (FWHM) of the PL, with smaller FWHM indicative of high optical quality. As can be seen in Supplementary Figure 3 (d), the PL exhibits FWHM as low as 35 meV, which is comparable to or even sharper than recent reports on exfoliated [3] as well as CVD synthesized WS_2 [4]. The corresponding Raman spectra measured at each site are shown in Supplementary Figure 3 (b). The E^1_{2g} and A_{1g} peaks, corresponding to the in-plane and out-of-plane Raman modes, are located at 357.5 cm^{-1} and 419 cm^{-1} respectively. The shoulder at 350 cm^{-1} indicates the longitudinal acoustic mode at the M point, LA(M). The separation of the E^1_{2g} and A_{1g} peaks is 61.5 cm^{-1}, which agrees with literature values for the peak separation for monolayer WS_2. Bilayer separation is reported to be 64 cm^{-1} [5].

Supplementary Note 4: Optical characterization of monolayer MoS_2 films

Large area optical images of the MoS_2 films are shown in Supplementary Figure 4. The bare substrate (top left corner), regions of monolayer growth (light purple) and multilayer growth (dark purple) are clearly visible. Supplementary Figure 4 (b) shows the border region between the bare substrate and monolayer growth. Detailed optical images of the monolayer area shows that the amount of multilayer content, as indicated by darker spots in the otherwise continuous background (Supplementary Figure 4c) increases towards the continuous multilayer areas.

Supplementary Note 5: Photoluminescence and Raman spectroscopy of the MoS_2 film

Room temperature PL of the MoS_2 film is measured using a 552 nm excitation laser and Raman spectra are acquired using a 488 nm excitation laser (Supplementary Figure 5). The Raman peak separation of the E^1_{2g} and A_{1g} peaks for MoS_2 is measured to be 20 cm^{-1}, which is typical for monolayer CVD MoS_2 [6]. Reports in the literature range from 18-20 cm^{-1}; the variation is attributed to strain and differences in the doping level. Exfoliated monolayer MoS_2 is reported to have a peak separation of 18 cm^{-1} [7], with multilayer MoS_2 ranging from 22 cm^{-1} (bilayer) to 25 cm^{-1} (bulk) [7]. The PL spectra of MoS_2 exhibit a single bright peak (see Supplementary Figure 5a). Consistent FWHM of 44 meV (Supplementary Figure 5d) and peak emission at ~ 1.84 eV are deduced from fits to the PL.

Supplementary References

[1] McCreary, K. M., Hanbicki, A. T., Jernigan, G. G., Culbertson, J. C., Jonker, B. T. Synthesis of large-area WS_2 monolayers with exceptional photoluminescence. *arXiv:*1512.00264*, in press*

[2] Ling, X., Lee, Y.-H., Lin, Y., Fang, W., Yu, L., Dresselhaus, M. S., Kong, J. Role of the seeding promoter in MoS_2 growth by chemical vapor deposition. *Nano Lett.* **14**, 464-472 (2014).

[3] Zhao, W., Ghorannevis, Z., Chu, L., Toh, M., Kloc, C., Tan, P.-H., Eda, G. Evolution of electronic structure in atomically thin sheets of WS_2 and WSe_2. *ACS Nano* **7**, 791-797 (2013).

[4] Peimyoo, N., Shang, J., Cong, C., Shen, X., Wu, X., Yeow, E. K. L., Yu, T. Nonblinking, intense two-dimensional light emitter: monolayer WS_2 triangles. *ACS Nano* **7**, 10985-10994 (2013).

[5] Berkdemir, A. *et al.* Identification of individual and few layers of WS_2 using Raman spectroscopy. *Nat. Sci. Reports* **3**:1755 (2013).

[6] Zhan, Y., Liu, Z., Najmaei, S., Ajayan, P. M., Lou, J. Large-area vapor-phase growth and characterization of MoS_2 atomic layers on a SiO_2 substrate. *Small* **8**, 966-971 (2012).

[7] Lee, C., Yan, H., Brus, L. E., Heinz, T. F., Hone, J., Ryu, S. Anomalous lattice vibrations of single and few-layer MoS_2. *ACS Nano* **4**, 2695-2700 (2010).
